# Long-Term Histologic Changes in Nasal Mucosa after Total Laryngectomy

**DOI:** 10.1155/2010/137128

**Published:** 2010-04-06

**Authors:** Çiğdem Tepe Karaca, Erdoğan Gültekin, M. Kürşat Yelken, Ayşenur Akyıldız İğdem, Mehmet Külekçi

**Affiliations:** ^1^Department of Otorhinolaryngology/Head and Neck Surgery, Haydarpaşa Numune Education and Research Hospital, Istanbul, Turkey; ^2^Department of Otorhinolaryngology/Head and Neck Surgery, Namık Kemal University, Tekirdağ, Turkey; ^3^Department of Otorhinolaryngology/Head and Neck Surgery, Gaziosmanpasa University, Tokat 60100, Turkey; ^4^Department of Pathology, Taksim Education and Research Hospital, Istanbul, Turkey; ^5^Department of Otorhinolaryngology/Head and Neck Surgery, Taksim Education and Research Hospital, Istanbul, Turkey

## Abstract

*Objective*. To determine the long-term histopathologic changes in nasal mucosa and the relationship between progression of the histopathologic changes and the duration without air current stimulation. *Material and Method*. Biopsies were taken from the inferior turbinates of 11 laryngeal cancer patients after total laryngectomy. Specimens were stained with hematoksilen-eosin and several histopathologic parameters were examined under light microscopy. *Results*. All of the patients demonstrated at least one histopathologic abnormality (100%, *n* = 11). Goblet destruction and stromal fibrosis were the most common findings (81%, *n* = 9), followed by focal epithelial atrophy and subepithelial seromusinous gland destruction (45%, *n* = 5), neovascularization and congestion (36%, *n* = 4), complete epithelial atrophy and mixoid degeneration (27%, *n* = 3). According to the duration between laryngectomy and biopsy, patients were grouped in to three: group 1; less than 12 months (36%, *n* = 4), group 2; 12–36 months (18%, *n* = 2), and group 3; more than 36 months (45%, *n* = 5). Only congestion was found to be decreased as the duration increased (*P* < .005). *Conclusion*. In laryngeal cancer patients histopathologic changes occur in nasal mucosa eventuate due to the cessation of air current stimulation, however there was no relation between progression of the histopathologic findings and the duration of cessation.

## 1. Introduction

The nose is a part of the airway system which is most easily accessible for morphological and pathophysiological evaluation of changes occurring as a response to various stimuli. After total laryngectomy in advanced laryngeal cancer patients, upper airway cannot do its physiological functions because upper and lower airways are separated [1]. The nose warms, cleans, and humidifies the inspired air. The absence of a physiological air flow stimulus after total laryngectomy leads to clinical, cytological, and histological changes of the nasal mucosa. The nasal mucosa was found to become thinner, and its color was observed to be changed over time [2–5]. Reduction of blood flow, deteriorated sense of smell and taste, changes in mucociliary transport and nasal flora are the other alterations [6, 7]. 

In this paper we aimed to determine the long term histopathological changes in nasal mucosa in total laryngectomized patients breathing through tracheastoma instead of the nose. Relationship between the duration of air cessation and the progression of histopathologic findings were also investigated. 

## 2. Material and Method

Eleven postoperative total laryngectomy patients were studied at Taksim Education and Research Hospital in Istanbul. All patients, who were asked to participate, agreed to take part. The study protocol was approved by the Ethical committee of Taksim Education and Research Hospital. Local anaesthesia was used to permit taking enough tissue for proper morphological analysis. For local anaesthesia 2% lidocaine HCl, 0.125% epinefrine solution (Jetokain, Adeka) injected into the mucosa of the inferior turbinate. Biopsy specimens were taken from the lower edge of the inferior turbinate and fixed in 10% buffered formaldehyde. The samples were dehydrated with increasing concentrations of alcohol and embedded in paraffin blocks. Serial 3-4 *μ*m thick tissues were cut at a plane perpendicular to the mucosal surface. Sections of the inferior turbinate were stained with hematoxylin-eosin and examined by Leica DMLS light microscope (Leica Microsystems, Wetzlar, Germany).

## 3. Results

All patients included in the study were male, and the average age was 58. All of the patients previously underwent total laryngectomy due to advanced stage laryngeal squamous cell carcinoma. Six patients had postoperative radiotherapy (45–70 Gy). 

**Figure 1:**
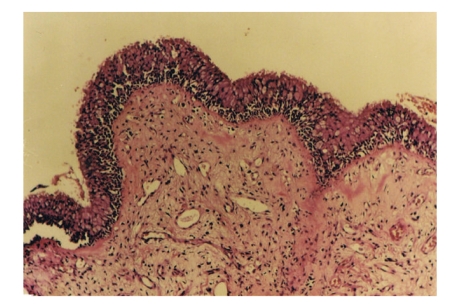
A light microscopic section of a nasal mucosa stained with hematoxylin-eosin. A normal nasal epithelium consisting of ciliated cells, goblet cells, and brush border cells. Below the basement membranes are glands and vessels.

**Figure 2:**
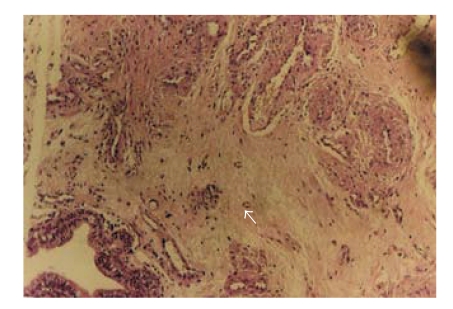
Myxoid degeneration in the stroma (hematoxylin-eosin, original magnification ×10).

**Figure 3:**
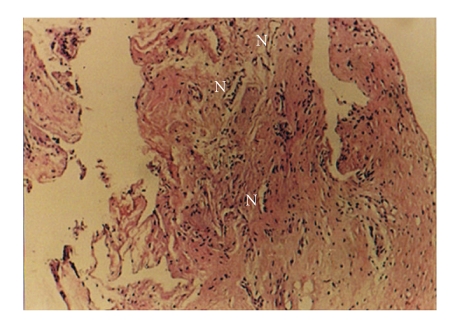
Neovascularization showed by letter N (hematoxylin-eosin, original magnification ×10).

**Figure 4:**
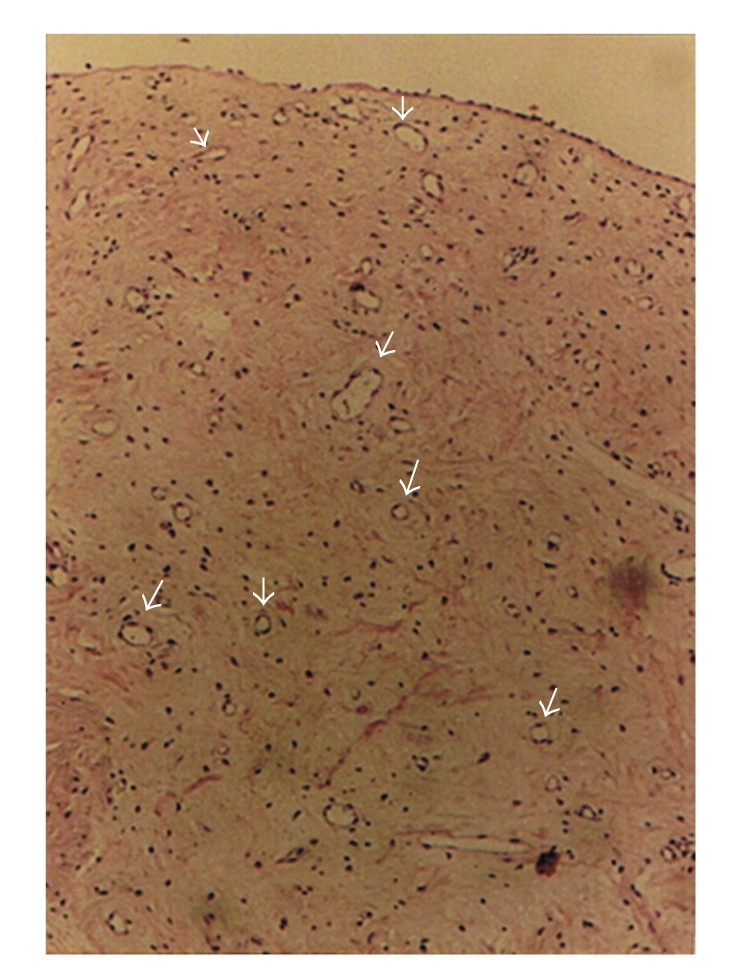
Neovascularization showed by arrow heads (hematoxylin-eosin, original magnification ×10).

**Figure 5:**
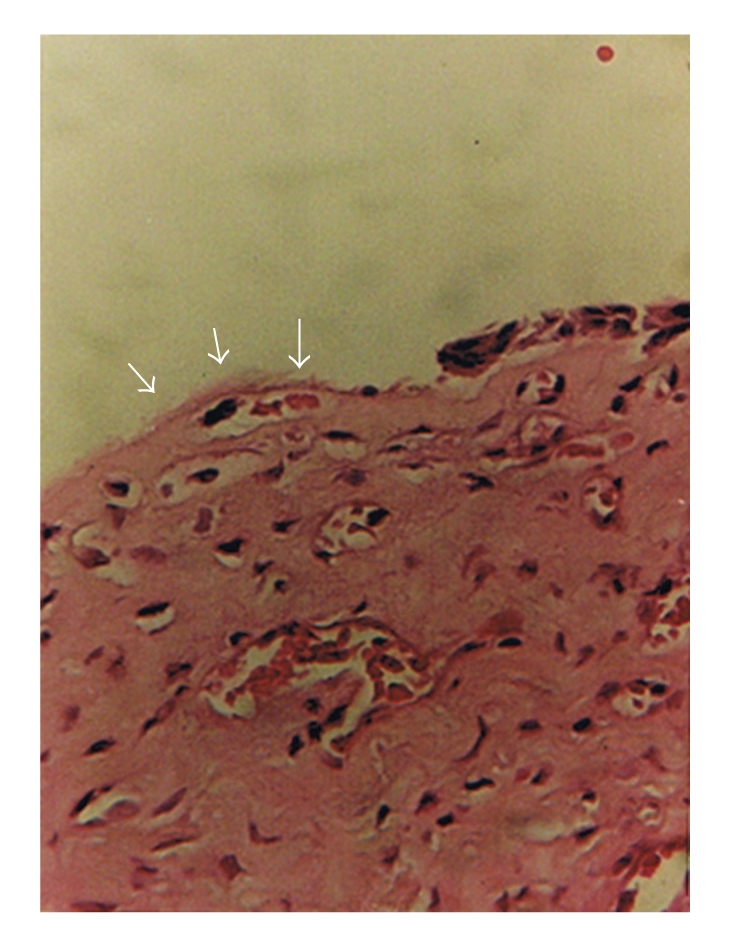
A section of the inferior turbinate showing atrophy of the epithelium by arrow heads (hematoxylin-eosin, original magnification ×40).

Atrophy in cilindiric epithel (focal or total), destruction of goblet cells, subepithelial glands and cilia, fibrosis of stroma, neovascularization, congestion and myxoid degeneration of the stroma were detected (Figures 1, 2, 3, 4, and 5). All of the patients (100%, *n* = 11) demonstrated at least one alteration in one of the above mentioned parameters (Table 1). The most frequently observed findings were goblet cell and stroma destruction (81%, *n* = 9) and the less frequent findings were total atrophy and myxoid degeneration of stroma (27%, *n* = 3); see Table 1. 

The average postoperative time until taking the biopsies was 31 months (minimum 6 months, maximum 84 months). Patients were divided into three groups according to biopsy time. The first group included four patients (36%) that biopsies were taken in the first postoperative year. Second group included two patients (18%), and biopsies were taken between the first and third years after total laryngectomy. Biopsies of five patients were taken after 36 months (45%). Statistically there was no significant difference when the histopathologic changes were compared among groups, except for congestion (*P* < .005); see Table 2. Congestion reduced significantly in time (*P* < .005); see Table 2.

## 4. Discussion

Treatment of advanced stages cancers of the larynx requires total laryngectomy. The continuity of respiratory airway is interrupted by total laryngectomy. In these patients, respiratory system begins from tracheostoma. The anatomical changes resulting from total laryngectomy have a serious impact on several important physical functions and thus on the patients quality of life. The laryngectomy patient provides us a convenient model to study nasal mucosal reaction to functional inactivity. When the laryngectomized patients examined rhinoscopically the nasal mucosa was found to become thinner and its color to be changed over time [8]. Mucosa was histologically examined to determine the response of the nasal cavity and six to twelve months after surgery the smear contained little mucus and cellular elements were found to be considerably decreased [9]. Nuclei of the cells were slightly enlarged, their structures were erased, and the membrane and chromatin distribution became hardly visible [9]. During a 1–5 year postlaryngectomy period, neither mucus nor goblet cells were observed. Low epithelial cells with or without cilia were frequently detected. Nasal biopsies showed that each patient had at least one change in histopahologic parameters. In eight patients (73%) atrophy of the epithelium was observed and five of them (46%) were focal atrophy. In other words the epithelium reduced to a single layer or the continuity ruined. Epithelium was preserved in three patients. 

Due to lack of physiological irritation of the mucosa by the air current, the multilayered cylindrical epithelium with cilia changes and goblet cells disappeared [9]. In our study, 81% of the patients' goblet cells disappeared. Also, 54% of the patients' ciliated cells were found to be destroyed. Little or no mucous was visible on the epithelium surface as a consequence of the decrease in mucus producing cells. 

Reduction in the amount of secretion or a loss of humidity at the mucosal surface would tend to cause destruction of the ciliated cells. In a study that nasal function was experimentally cut off by surgical obstruction of both nasal openings; epithelium reduced to a single layer of cubical cells [10]. Lamina propria at some sites was swollen due to connective tissue expansion, capillaries were dilated, and thickening of some blood vessel walls was observed [10]. In our study, lamina propria became wider due to fibrosis in nine patients (81%) and congestion in four (36%). Neovascularization occurred in four patients (36%). The reduction in the air flow through the nose is responsible for such findings. We thought that by cessation of nasal air current, histopathologic changes might progress over time, however, our data was unable to confirm this. Mucosal changes occurred soon after surgery and did not modify over time. The reason may be that the observed histopathologic changes are already the last stage of mucosal, goblet cellular, ciliar, and glandular destruction, stromal fibrosis, and atrophy. 

There is a potential relationship between the histological changes in nasal mucosa and the qualitative/quantitative changes in nasal dysfunction in laryngectomized patients. Due to reduction of blood flow, changes in mucociliary transport and deteriorated sense of smell and taste have been previously reported [6, 7]. With the observed histological findings some functional changes also occurred in our patients such as dryness in the nose, smelling, and taste problems. However, we did not routinely perform objective taste and smell tests. Based on our results and the previous data, rehabilitation programs concerning the problems in olfaction and taste for laryngectomized patients should be developed. Artificial stimulation of the respiratory mucous by air stream may protect and normalize the epithelium but the question whether it would be a way to improve the capacity of smelling of the patients or not, still needs to be investigated. 

## 5. Conclusion

After total laryngectomy, histopathologic changes occur in nasal mucosa. Pseudostratified columnar ciliated epithelium changes, decrease in goblet cells and submucosal glands, fibrosis in stroma, myxoid degeneration, neovascularization, and congestion in lamina propria at some sites are detected in histopathologic examination. The changes in the nasal mucosa after airflow cessation are dynamic and require months to equilibrate. Stimulation of the respiratory mucous membrane by air stream may be important factor for normal functioning of the ciliary apparatus, glandular, and vascular elements of the nasal mucosa.

## Figures and Tables

**Table 1:** The numbers of patients according to histopathologic changes.

Histopathologic changes	Patients
Destruction of goblet cells	81% (*n* = 9)
Fibrosis in stroma	81% (*n* = 9)
Destruction of cilia	54% (*n* = 6)
Destruction of glands	45% (*n* = 5)
Focal atrophy	45% (*n* = 5)
Total atrophy	27% (*n* = 3)
Neovascularization	36% (*n* = 4)
Congestion	36% (*n* = 4)
Myxoid degeneration	27% (*n* = 3)

**Table 2:** The comparison of duration of time between laryngectomy and biopsy. Group1, less than 12 months, group 2, 12–36 months, group 3, more than 36 months. *In group 3, three patients have total atrophy; one patient has focal atrophy. In the other groups atrophy is focal. **P < .005.

Histopathologic changes	Groups		
	Group 1 (*n* = 4)	Group 2 (*n* = 2)	Group 3 (*n* = 5)
Destruction of goblet cells	%50 (*n* = 2)	%100 (*n* = 2)	%100 (*n* = 5)
Fibrosis in stroma	%100 (*n* = 4)	%100 (*n* = 2)	%60 (*n* = 3)
Destruction of cilia	%50 (*n* = 2)	%50 (*n* = 1)	%60 (*n* = 3)
Destruction of glands	%25 (*n* = 1)	%25 (*n* = 1)	%60 (*n* = 3)
Atrophy	%75 (*n* = 3)	%50 (*n* = 1)	%80 (*n* = 4)*
Neovascularization	%50 (*n* = 2)	—	%40 (*n* = 2)
Congestion	%75 (*n* = 3)**	%50 (*n* = 1)**	—
Mixoid degeneration	%25 (*n* = 1)	%50 (*n* = 1)	%20 (*n* = 1)
